# A Role for Nuclear F-Actin Induction in Human Cytomegalovirus Nuclear Egress

**DOI:** 10.1128/mBio.01254-16

**Published:** 2016-08-23

**Authors:** Adrian R. Wilkie, Jessica L. Lawler, Donald M. Coen

**Affiliations:** Department of Biological Chemistry and Molecular Pharmacology and Committee on Virology, Harvard Medical School, Boston, Massachusetts, USA

## Abstract

Herpesviruses, which include important pathogens, remodel the host cell nucleus to facilitate infection. This remodeling includes the formation of structures called replication compartments (RCs) in which herpesviruses replicate their DNA. During infection with the betaherpesvirus, human cytomegalovirus (HCMV), viral DNA synthesis occurs at the periphery of RCs within the nuclear interior, after which assembled capsids must reach the inner nuclear membrane (INM) for translocation to the cytoplasm (nuclear egress). The processes that facilitate movement of HCMV capsids to the INM during nuclear egress are unknown. Although an actin-based mechanism of alphaherpesvirus capsid trafficking to the INM has been proposed, it is controversial. Here, using a fluorescently-tagged, nucleus-localized actin-binding peptide, we show that HCMV, but not herpes simplex virus 1, strongly induced nuclear actin filaments (F-actin) in human fibroblasts. Based on studies using UV inactivation and inhibitors, this induction depended on viral gene expression. Interestingly, by 24 h postinfection, nuclear F-actin formed thicker structures that appeared by super-resolution microscopy to be bundles of filaments. Later in infection, nuclear F-actin primarily localized along the RC periphery and between the RC periphery and the nuclear rim. Importantly, a drug that depolymerized nuclear F-actin caused defects in production of infectious virus, capsid accumulation in the cytoplasm, and capsid localization near the nuclear rim, without decreasing capsid accumulation in the nucleus. Thus, our results suggest that for at least one herpesvirus, nuclear F-actin promotes capsid movement to the nuclear periphery and nuclear egress. We discuss our results in terms of competing models for these processes.

## IMPORTANCE

The mechanisms underlying herpesvirus nuclear egress have not been fully determined. In particular, how newly assembled capsids move to the inner nuclear membrane for envelopment is uncertain and controversial. In this study, we show that HCMV, an important human pathogen, induces actin filaments in the nuclei of infected cells and that an inhibitor of nuclear F-actin impairs nuclear egress and capsid localization toward the nuclear periphery. Herpesviruses are widespread pathogens that cause or contribute to an array of human diseases. A better understanding of how herpesvirus capsids traffic in the nucleus may uncover novel targets for antiviral intervention and elucidate aspects of the nuclear cytoskeleton, about which little is known.

## INTRODUCTION

Herpesviruses execute important steps of their replication cycles in the host cell nucleus. Among these steps is viral DNA synthesis, which occurs in discrete structures called replication compartments (RCs). Assembled capsids are then packaged with DNA before they translocate from the nucleus to the cytoplasm in a process called nuclear egress. To facilitate these steps, herpesviruses impart profound changes to host nuclear architecture, including the formation and expansion of RCs, partitioning of host chromatin, and disruption of the nuclear lamina (1).

Human cytomegalovirus (HCMV) is a betaherpesvirus that is an important pathogen in immunocompromised and immune-naive individuals (2). During infection with HCMV, viral DNA synthesis occurs away from the nuclear rim at the periphery of RCs (3–6). The presence of capsid and terminase proteins in RCs suggests that capsid assembly and packaging are spatially coordinated with DNA synthesis within the nuclear interior (7–12). Assembly and packaging are followed by nuclear egress, which includes movement of capsids to the nuclear rim, disruption of the nuclear lamina, capsid envelopment at the inner nuclear membrane (primary envelopment), and finally deenvelopment at the outer nuclear membrane (13, 14). While much progress has been made toward understanding events at the nuclear rim (13, 15–23), very little is known about earlier steps of nuclear egress. Specifically, how HCMV capsids move from RCs to the nuclear periphery for primary envelopment is unknown.

Several studies have suggested roles for nuclear actin filaments (F-actin) during alphaherpesvirus infection. One study showed that herpes simplex virus 1 (HSV-1) RCs move in a manner that was antagonized by inhibitors of F-actin and myosin (24). It has also been reported that infection with pseudorabies virus (PRV) or HSV-1 induces nuclear F-actin in neuronal cells and that capsids colocalize with actin filaments and a myosin motor protein (25). Using particle tracking analysis, another group found directed intranuclear movements of HSV-1 capsids that were antagonized by ATP depletion or by inhibitors of myosin and F-actin (26). Despite these reports, the notion that a nuclear F-actin-based mechanism facilitates herpesvirus capsid motility has recently been challenged by Bosse et al. (27, 28). This group was unable to visualize actin filaments in the nuclei of murine embryonic fibroblasts (MEFs) infected with PRV, HSV-1, mouse cytomegalovirus (MCMV), and murine gammaherpesvirus 68 (MHV-68) (27). They also reported that alphaherpesvirus (PRV and HSV-1) infection alters chromatin domains so that capsids can efficiently move by diffusion rather than by a directed mechanism to the nuclear periphery (28). Regardless, whether nuclear F-actin is induced or has any role during HCMV infection, or whether F-actin plays a role in nuclear egress *per se* for any herpesvirus, has not been reported. Further investigation into the importance of the nuclear cytoskeleton for herpesvirus infection is important not only for virology but also for cell biology, as the functions of nuclear F-actin remain largely unexplored.

In the present study, we began by asking whether nuclear actin filaments are present in HCMV-infected cells. We observed a striking induction of nuclear F-actin in HCMV-, but not HSV-1-, infected human fibroblasts. Induction of nuclear F-actin began early in infection and relied on viral gene expression. Examination of nuclear F-actin localization relative to RCs revealed that filaments localized along the periphery of RCs and extended between the RC periphery and the nuclear rim. Crucially, an inhibitor that depolymerizes nuclear F-actin caused defects in the production of infectious virus, capsid accumulation in the cytoplasm, and capsid localization toward the nuclear periphery. We discuss the implications of our results for competing models by which nuclear F-actin would abet movement of capsids from RCs to the nuclear rim.

## RESULTS

### Induction of nuclear F-actin during HCMV infection.

**FIG 1:**
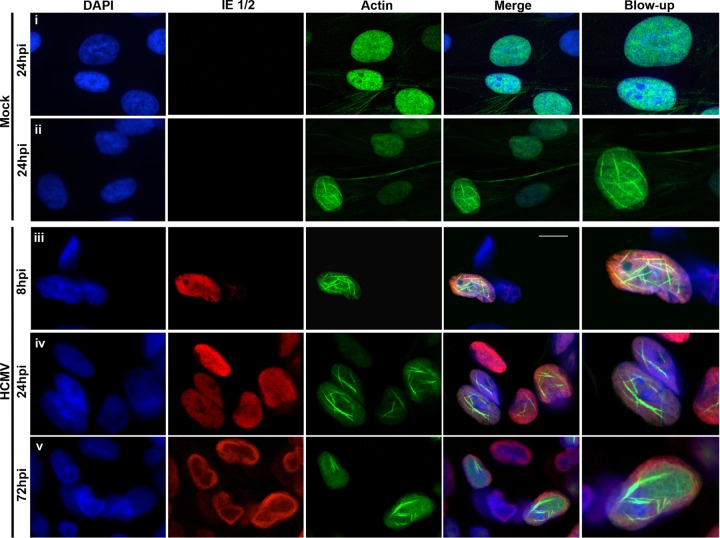
HCMV induces nuclear F-actin. HFFs stably expressing LifeAct-GFP-NLS (green) were either mock infected (rows i and ii) or infected (rows iii to v) with WT HCMV (MOI of 1). Cells were fixed at the indicated time points, stained with an anti-IE 1/2 antibody (red) and DAPI (blue), and imaged with spinning-disk confocal microscopy. Images are single Z-sections. Bar, 10 µm.

To determine whether nuclear F-actin is present in HCMV-infected cells, we utilized LifeAct-green fluorescent protein (GFP), an actin-binding peptide that has been extensively used to visualize F-actin and does not interfere with its dynamics *in vitro* or *in vivo* (29). To more easily discern filaments in the nucleus, we generated human foreskin fibroblasts (HFFs) stably expressing LifeAct-GFP fused to a nuclear localization signal (LifeAct-GFP-NLS) that has previously been used to visualize nuclear F-actin (30, 31). LifeAct-GFP-NLS-expressing HFFs were either mock infected or infected with wild-type (WT) HCMV (multiplicity of infection [MOI] of 1). Cells were stained with 4′,6-diamidino-2-phenylindole (DAPI) to visualize nuclei, and infected nuclei were additionally marked by staining with antibodies against HCMV IE 1/2 proteins. During mock infection, the vast majority of cells (93%, *n* = 118) contained diffuse LifeAct-GFP-NLS primarily, but not exclusively, in the nucleus, with no nuclear F-actin apparent (Fig. 1, row i). However, a small minority of cells (7%) displayed a network of what appeared to be individual nuclear actin filaments (Fig. 1, row ii). These results are consistent with previous reports that nuclear F-actin can form in some uninfected cells (30–33). In contrast, nuclear F-actin was induced in LifeAct-GFP-NLS-expressing HFFs infected with HCMV starting around 6 h postinfection (hpi) (see Movie S1 in the supplemental material), with 88% (*n* = 25) of infected cells at 8 hpi, 92% (*n* = 110) at 24 hpi, and 74% (*n* = 86) at 72 hpi containing nuclear F-actin (examples in Fig. 1, rows iii to v). Interestingly, by 24 hpi, most filaments formed thicker structures (Fig. 1A, rows iv and v; also see Movie S1). To better resolve these thicker structures, we utilized super-resolution three-dimensional structured illumination microscopy (3D-SIM) and found what appeared to be bundled F-actin filaments at various orientations throughout the nuclear volume (see Movie S2). Finally, as it has been reported that alphaherpesvirus capsids colocalize with virus-induced nuclear F-actin (25), we investigated whether an HCMV capsid protein (major capsid protein [MCP]) would colocalize with nuclear actin filaments. At 72 hpi, we observed colocalization of actin filaments and MCP in the nucleus (see Fig. S1).

**FIG 2:**
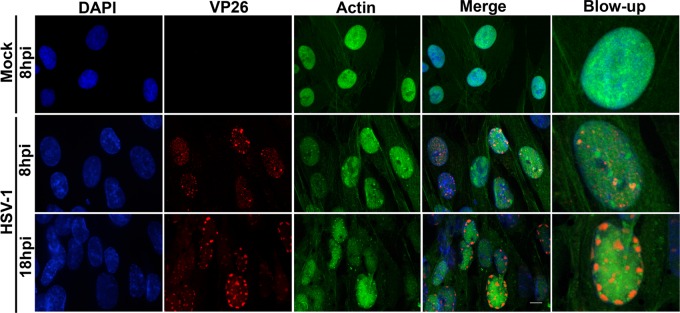
Nuclear F-actin visualization in HSV-1-infected cells. HFFs stably expressing LifeAct-GFP-NLS (green) were either mock infected or infected with VP26-RFP (red) HSV-1 (MOI of 3). At the time points indicated, cells were fixed, stained with DAPI (blue), and imaged with spinning-disk confocal microscopy. Images are single Z-sections. Bar, 10 µm.

We wondered whether HSV-1 would similarly induce nuclear F-actin in HFFs. To explore this possibility, LifeAct-GFP-NLS-expressing HFFs were infected with HSV-1 encoding the VP26 capsid protein fused to red fluorescent protein (VP26-RFP; MOI of 3), thus marking infected cells. We observed that the vast majority of HSV-1-infected cells displayed only diffuse LifeAct-GFP-NLS in the nucleus at both 8 hpi (94%, *n* = 84) and 18 hpi (87%, *n* = 93) (Fig. 2). In the small number of HSV-1-infected cells that did contain nuclear F-actin, filaments appeared shorter and thinner than in HCMV-infected cells and did not colocalize meaningfully with VP26-RFP (see Fig. S2 in the supplemental material). Thus, HSV-1 and HCMV differ in their capacity to induce nuclear F-actin in HFFs.

Herpesviruses are known to disrupt cytoplasmic F-actin stress fibers (34–36); thus, we asked whether the LifeAct-GFP-NLS induces nuclear F-actin by “dragging” monomeric G-actin into the nucleus. To examine this possibility, LifeAct-GFP-NLS-expressing HFFs were either mock infected or infected with WT HCMV, and lysates were separated into nuclear and cytoplasmic fractions. Using Western blot analysis, we confirmed successful fractionation based on the distribution of lamin B and glyceraldehyde-3-phosphate dehydrogenase (GAPDH) but were unable to detect an increase in either β-actin or LifeAct-GFP-NLS levels in the nucleus during infection, suggesting that HCMV likely induces nuclear F-actin from a preexisting pool of monomeric nuclear G-actin (see Fig. S3 in the supplemental material).

### Determinants of nuclear F-actin induction.

**FIG 3:**
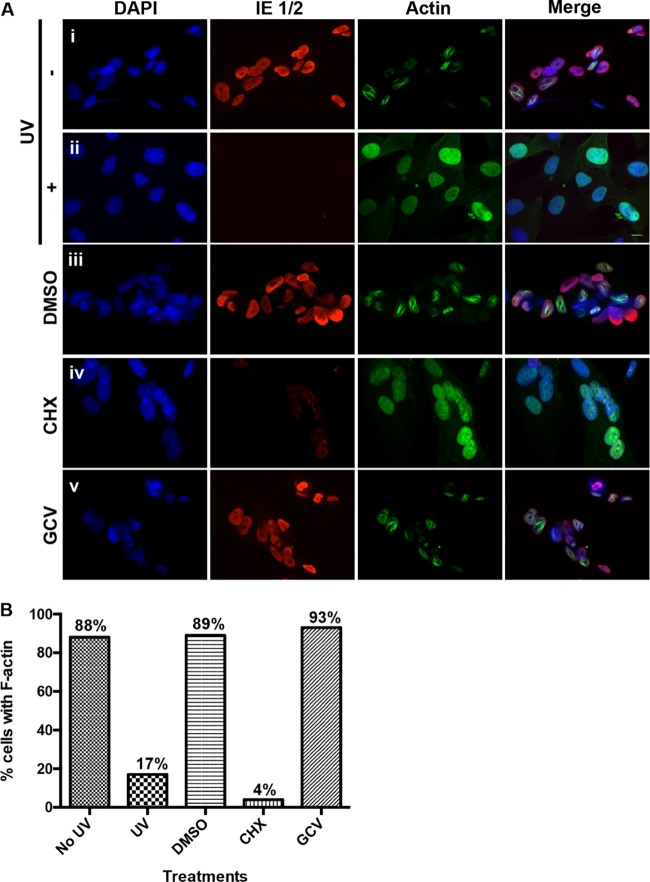
Determinants of nuclear F-actin induction. (A) Rows i and ii, HFFs stably expressing LifeAct-GFP-NLS (green) were infected with either UV-inactivated (+) or mock-UV-inactivated (−) WT HCMV (MOI of 1). At 24 hpi, cells were fixed, stained with an anti-IE 1/2 antibody (red) and DAPI (blue), and imaged with spinning-disk confocal microscopy. Rows iii to v, LifeAct-GFP-NLS-expressing HFFs were infected with WT HCMV (MOI of 1) and treated with cycloheximide (CHX), ganciclovir (GCV), or DMSO vehicle between 0 and 24 hpi. Cells were then fixed and processed as described above. Images are single Z-sections. Bar, 10 µm. (B) The percentage of cells with detectable filamentous LifeAct-GFP-NLS staining was quantified for each condition described above (no UV, *n* = 47; UV, *n* = 50; DMSO, *n* = 55; CHX, *n* = 48; GCV, *n* = 66).

Given that nuclear F-actin is induced starting around 6 h following HCMV infection, we hypothesized that either virion structural proteins and/or immediate early gene expression is responsible for induction. To discriminate between these possibilities, we infected LifeAct-GFP-NLS-expressing HFFs with UV-inactivated or mock-UV-inactivated WT HCMV (MOI of 1), fixed cells at 24 hpi, and stained cells with DAPI and with anti-IE 1/2 antibodies. Fluorescence microscopy revealed that UV inactivation substantially reduced IE 1/2 gene expression, as expected, and greatly reduced the percentage of cells containing nuclear F-actin, suggesting that functional viral genes are required for induction, while virion structural proteins are not sufficient for induction (Fig. 3A, rows i and ii, and B). Similarly, inhibition of protein synthesis with cycloheximide drastically reduced IE 1/2 gene expression and the percentage of cells displaying filaments compared to dimethyl sulfoxide (DMSO)-treated cells (Fig. 3A, rows iii and iv, and B). Conversely, cells treated with ganciclovir (GCV), an inhibitor of viral DNA synthesis, displayed a similar percentage of cells with nuclear F-actin as did DMSO-treated cells (Fig. 3A, rows iii and v, and B). The concentration of GCV used greatly reduced the expression of a late viral protein, confirming drug efficacy (see Fig. S4 in the supplemental material). Collectively, these results, coupled with our previous finding that induction occurs in most cells by 8 hpi, suggest that HCMV specifically induces nuclear F-actin through the expression of one or more immediate early or possibly early gene products.

### Nuclear F-actin localization relative to RCs.

**FIG 4:**
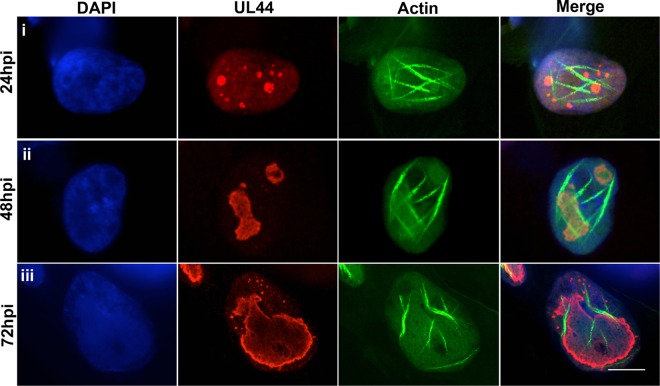
Nuclear F-actin localization relative to RCs. LifeAct-GFP-NLS (green)-expressing HFFs were infected with 44-F HCMV (MOI of 1), fixed at the indicated time points, stained with an anti-FLAG antibody (red) and DAPI (blue), and imaged with spinning-disk confocal microscopy. Images are single Z-sections. Bar, 10 µm.

Given the importance of RCs for HCMV replication and previous evidence that nuclear F-actin facilitates HSV-1 RC movement (24), we wondered how actin filaments would localize relative to RCs at different stages of HCMV infection. We therefore infected LifeAct-GFP-NLS-expressing HFFs with HCMV encoding a FLAG-tagged version of UL44 (44-F; MOI of 1), the viral DNA polymerase subunit that has previously been shown to concentrate at the periphery of RCs (3, 4). Cells were then fixed at 24, 48, and 72 hpi and stained with DAPI and an anti-FLAG antibody, and single optical sections were imaged using confocal microscopy. By 24 hpi, multiple small RCs formed within the nucleoplasm, as expected. In many cases, RCs were found adjacent to actin filaments (Fig. 4, row i). By later times postinfection (48 to 72 hpi), when RCs had merged and expanded, nuclear F-actin primarily localized along the periphery of RCs, with some extending between the RC periphery and the nuclear rim, and some orienting along the nuclear rim (Fig. 4, rows ii and iii; Fig. S5 in the supplemental material contains additional representative images and quantification of cells containing different filament orientations). These results raise the possibility of associations between nuclear actin filaments and events occurring at or near the RCs.

### Latrunculin A depolymerizes nuclear F-actin and causes defects in viral production and nuclear egress.

It has been proposed that HSV-1 hijacks nuclear F-actin for capsid trafficking during nuclear egress (26), but a role for F-actin in nuclear egress *per se* for any herpesvirus has not been shown. To investigate this issue, we began by testing whether various concentrations of two widely used inhibitors of F-actin polymerization, latrunculin A (LatA) and cytochalasin D (CytoD), antagonize nuclear F-actin in HCMV-infected HFFs expressing LifeAct-GFP-NLS. In these experiments, we infected cells with WT HCMV (MOI of 5) and at 72 hpi replaced the medium with medium containing LatA, CytoD, or vehicle control (DMSO) for 24 h before fixing cells for fluorescence microscopy. This allowed us to measure the effects of actin inhibitors specifically during peak hours of nuclear egress (72 to 96 hpi). We found that nuclear F-actin was present in most DMSO-treated LifeAct-GFP-NLS-expressing cells, as expected (see Fig. S6A, row i, in the supplemental material). While some nuclear F-actin was evident in cells treated with 4 µM LatA, all cells treated with 8 µM LatA exhibited diffuse GFP signal with little or no nuclear F-actin apparent (see Fig. S6A, rows ii and iii). Interestingly, even upon treatment with 4 µM CytoD (see Fig. S6A, row iv) or 8 µM CytoD (data not shown), some cells still displayed nuclear F-actin. We also noted that the nuclei of cells treated with all concentrations of LatA and CytoD were significantly smaller than those in DMSO-treated cells, consistent with what was reported with HSV-1 (see Fig. S6A, rows i to iv) (37). Furthermore, with all concentrations of LatA and CytoD tested, HFFs displayed a rounded morphology characteristic of cytoplasmic F-actin depolymerization (see Fig. S6B). Thus, CytoD failed to fully depolymerize nuclear F-actin despite exerting effects elsewhere in the cell. Collectively, these results indicate that a critical concentration of LatA is required to antagonize nuclear F-actin and that CytoD is a less efficacious antagonist.

**FIG 5:**
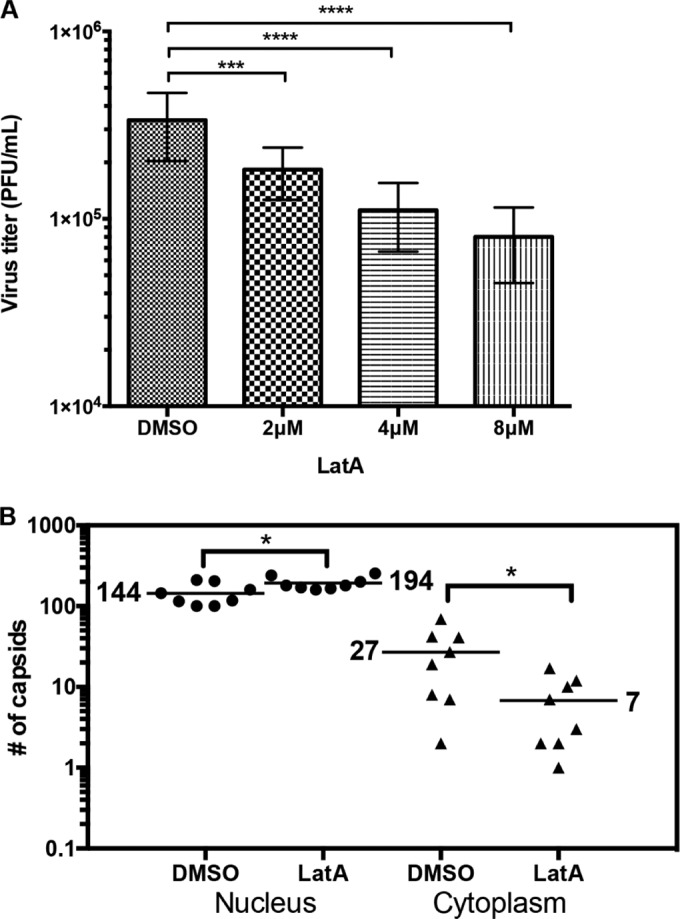
LatA causes defects in viral production and nuclear egress. (A) HFFs were infected with WT HCMV (MOI of 5). Medium was removed at 72 hpi and replaced with fresh medium containing the indicated concentrations of LatA or DMSO vehicle. At 96 hpi, medium was removed for titration to assess production of infectious virus. The graph shows averages ± standard deviations from 3 independent experiments. *P* values were calculated using one-way repeated-measures analysis of variance corrected for multiple comparisons using the Holm-Sidak test. ***, *P* ≤ 0.0003; ****, *P* ≤ 0.0001 (DMSO versus 2 µM LatA, *P* = 0.0003; DMSO versus 4 µM LatA, *P* < 0.0001; DMSO versus 8 µM LatA, *P* < 0.0001). (B) The cell monolayers from above treated with 8 µM LatA or DMSO were fixed and processed for EM, and capsids were counted in the nucleus and cytoplasm of whole-cell sections in 8 cells for each condition. The horizontal bars indicate the mean number of capsids for each condition. *P* values were calculated using the Mann-Whitney test; *, *P* ≤ 0.05 (*P* = 0.04).

We then sought to determine whether LatA treatment would impair HCMV replication and nuclear egress. Using the experimental protocol described above, we found that LatA caused a dose-dependent decrease in the production of infectious virus, with the highest defect (~5-fold) occurring with 8 µM drug (Fig. 5A). We also analyzed cells by electron microscopy (EM) to determine whether LatA treatment inhibits capsid accumulation in the cytoplasm. Notably, treatment with 8 µM LatA caused a 4-fold defect in the mean number of cytoplasmic capsids compared to DMSO-treated cells, which was statistically significant (*P* = 0.04). Importantly, there was no decrease in the mean number of nuclear capsids; in fact, there was a 26% increase, which was significant (*P* = 0.04) (Fig. 5B). There was no significant difference in total number of capsids (cytoplasmic and nuclear) between LatA-treated and DMSO-treated cells (*P* = 0.12). As an additional control, we tested whether LatA treatment affects RC formation. LifeAct-GFP-NLS-expressing HFFs infected with 44-F HCMV (MOI of 1) in the presence of 8 µM LatA between 0 and 48 hpi exhibited what appeared to be mature RCs in all cells analyzed (*n* = 10), suggesting that nuclear F-actin is not important for RC formation or expansion, at least at early stages of infection (see Fig. S7 in the supplemental material). Thus, our results suggest that nuclear F-actin depolymerization antagonizes the production of infectious virus, which is in large part due to specific inhibition of HCMV nuclear egress.

### Nuclear F-actin is important for capsid localization away from RC-like inclusions.

**FIG 6:**
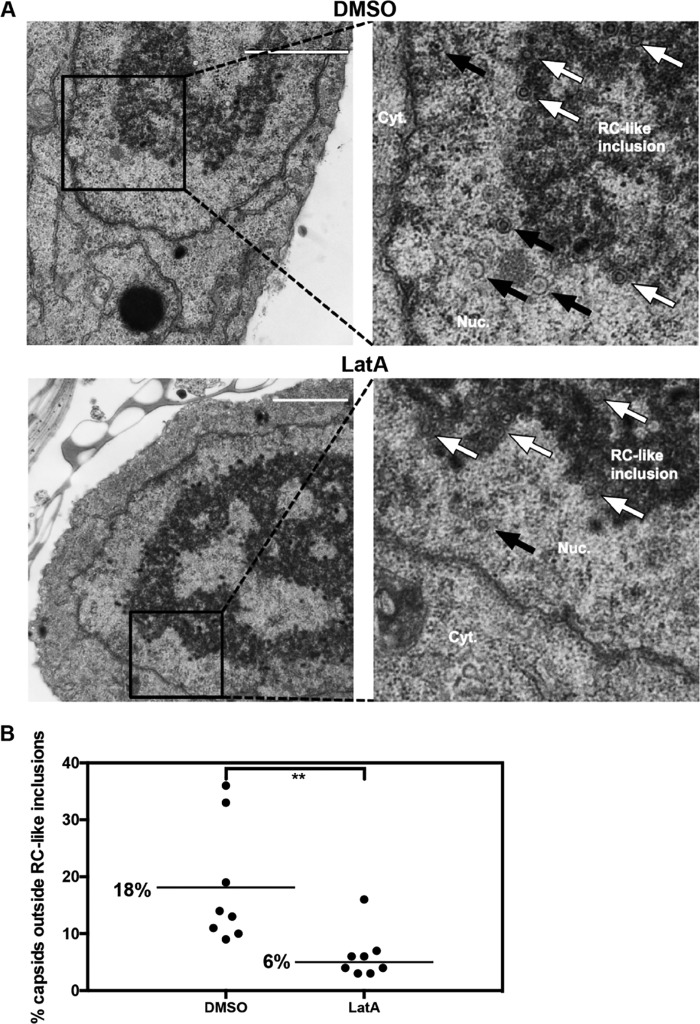
LatA inhibits capsid localization away from RC-like inclusions toward the nuclear periphery. The EM images of whole-cell sections of infected cells treated with LatA or DMSO vehicle control from Fig. 5 (8 cells for each condition) were analyzed for localization of capsids in or away from RC-like inclusions. (A) Representative nuclear sections for each condition. White arrows show examples of capsids in RC-like inclusions; black arrows show examples of capsids outside RC-like inclusions. Bar, 2 µm. (B) The percentage of capsids not associated with RC-like inclusions was calculated for each nucleus and plotted. Bars and the numbers alongside them indicate mean percentages of capsids. *P* values were calculated using the Mann-Whitney test; **, *P* ≤ 0.01 (*P* = 0.003).

To further interrogate the role of actin filaments in HCMV nuclear egress, we asked whether nuclear F-actin depolymerization would impair capsid localization away from the nuclear interior. EM analysis of HCMV-infected cells in the abovementioned experiments revealed the formation of capsid-containing electron-dense inclusions in the interior of the nucleoplasm that have been considered RCs (38, 39), which we term RC-like inclusions (Fig. 6A). We therefore calculated the percentage of capsids located outside these structures, and thus in closer proximity to the nuclear periphery, in cell sections from the above-described experiment (Fig. 6B). We found that 18% of capsids were present outside RC-like inclusions in DMSO-treated cells compared to 6% in LatA-treated cells (3-fold decrease), and this difference was highly significant (*P* = 0.003). We therefore infer that inhibition of actin polymerization reduces capsid localization away from RCs toward the nuclear periphery.

## DISCUSSION

How newly assembled herpesvirus capsids move to the nuclear periphery is poorly understood and controversial. In this study, we found that HCMV but not HSV-1 infection leads to a striking induction of nuclear actin filaments. Induction of nuclear F-actin began within 6 h following HCMV infection and relied on viral gene expression but not DNA replication. Interestingly, nuclear F-actin frequently formed thick structures adjacent to RCs. Later in infection, filaments were primarily localized along the RC periphery and between the RC periphery and the nuclear rim, where they colocalized with capsid protein. Importantly, treatment with LatA resulted in depolymerization of nuclear F-actin and defects in viral production, capsid accumulation in the nucleus, and intranuclear capsid localization away from RCs toward the nuclear rim. We discuss each of these aspects of our results and possible models by which nuclear F-actin could facilitate capsid movement from RCs to sites of primary envelopment during HCMV nuclear egress.

### Nuclear F-actin induction.

Our finding of nuclear F-actin induction during HCMV infection of HFFs using LifeAct-GFP-NLS is consistent with previous results with PRV and HSV-1 infection of mouse neurons and PRV infection of a swine epithelial cell line (25). In those cases, induction of nuclear F-actin was detected using serial block-face scanning electron microscopy or phalloidin staining (25). Recently, however, the same group found that MEFs expressing LifeAct-GFP (without an NLS) that were infected with PRV, HSV-1, MCMV, or MHV-68 did not display nuclear F-actin (27), which is consistent with our inability to detect nuclear F-actin induction in the majority of HSV-1-infected HFFs. Regardless, certain alpha- and betaherpesviruses are capable of inducing nuclear F-actin, at least in some cell types. Differences among viruses and cell types may well explain discrepancies in reports addressing nuclear F-actin induction.

We also emphasize that it can be difficult to detect nuclear F-actin using certain techniques that also stain cytoplasmic actin, as reported by others (30, 32). LifeAct-GFP-NLS affords the advantage of concentrating actin-binding probe in the nucleus, which enhances nuclear F-actin visualization and minimizes staining in the cytoplasm.

We observed what appeared to be a network of individual nuclear actin filaments in a small population of mock-infected cells and in the majority of HCMV-infected cells starting early in infection, whereas later in infection many filaments appeared thicker. Our super-resolution microscopy results suggest that these thick structures are bundles of individual filaments. The exact mechanism by which HCMV infection induces nuclear F-actin and these thicker structures is unknown. Nevertheless, our results suggest that one or more viral immediate early or possibly early gene products promote nuclear F-actin polymerization, as induction occurs starting around 6 hpi and relies on viral gene expression but not DNA replication. These viral gene products might induce nuclear F-actin directly or by effects on host protein expression or function. Cellular protein candidates that could be affected by HCMV infection include mDia formin proteins, which are important for nuclear F-actin induction upon serum stimulation and cell spreading (30, 31).

Finally, we note that induction of nuclear F-actin is not confined to herpesviruses, as baculovirus has been shown to use nuclear F-actin polymerization for propulsion in a manner similar to *Listeria monocytogenes* cytoplasmic motility (41).

### LatA inhibition of nuclear F-actin.

Even at concentrations and times of exposure where both LatA and CytoD caused cell rounding, indicative of cytoplasmic actin depolymerization, LatA was more efficacious than CytoD at antagonizing HCMV-induced nuclear F-actin and impaired HCMV production and nuclear egress. This finding is reminiscent of the previous report that LatA, but not CytoD, inhibits directed intranuclear movements of HSV-1 capsids (26). LatA antagonizes F-actin by preventing the formation of new filaments, while CytoD binds F-actin and prevents additional polymerization (42, 43). It has been speculated that nuclear F-actin may adopt a structure distinct from cytoplasmic F-actin (26, 44), which may account for the different efficacies of the two drugs. Alternatively, CytoD may not reach its nuclear target as effectively as LatA.

Using LifeAct-GFP without an NLS, Bosse et al. reported that nuclear actin rods formed in PRV-infected MEFs after 1 h of treatment with LatA but not CytoD (27). Using LifeAct-GFP-NLS, we found that treatment with a higher concentration of LatA for a longer duration depolymerized HCMV-induced nuclear F-actin, in keeping with its known activity. It was also previously reported that neither LatA nor CytoD impairs HSV-1 virus yield (37). However, the concentrations of LatA (1 to 3 µM) used in that study were less than the concentration (4.7 µM) used to inhibit directed movements of HSV-1 capsids (26) and less than the concentration required to depolymerize most HCMV-induced nuclear F-actin.

### Nuclear F-actin and RCs.

We observed that HCMV-induced nuclear F-actin was often located adjacent to RCs. Studies on multiple DNA viruses have suggested involvement of nuclear F-actin in RC movement and function (24, 45). As we found that depolymerization of nuclear F-actin with LatA did not affect HCMV RC formation and did not reduce nuclear capsid accumulation, our data indicate that, at least under the conditions of our studies, nuclear F-actin is not crucial for capsid assembly or certain steps preceding it. Thus, the association of F-actin with HCMV RCs seems more likely to play a role in subsequent steps, such as nuclear egress.

### Importance of F-actin for capsid localization from RCs to the nuclear periphery.

Using EM, we found that LatA treatment caused HCMV capsids to accumulate in RC-like inclusions away from the nuclear rim. It has been reported that inhibition of nuclear F-actin affects capsid distribution in PRV-infected nuclei (25). In that case, depolymerization of nuclear F-actin disrupted the localization of capsids to small capsid-rich foci, leading these authors to speculate that filaments serve as a scaffold for capsid assembly. However, we did not observe a negative effect of LatA on capsid assembly. We (this study) and others (39) have observed that HCMV capsids frequently associate with inclusions in the nuclear interior that are highly reminiscent of RCs visualized with fluorescence microscopy, suggesting that capsid assembly occurs in RCs. As we found that LatA treatment led to a decrease in the percentage of capsids located outside these inclusions and that capsid protein colocalizes with actin filaments in the nucleus, we suggest that nuclear F-actin mediates the localization of newly assembled capsids from RCs toward the nuclear periphery. Moreover, as the magnitudes of the effects of LatA on intranuclear capsid localization, nuclear egress, and viral production were similar, it seems likely that the major role for nuclear F-actin during HCMV infection is in movement of capsids toward the nuclear periphery during nuclear egress.

### Models.

Our data are consistent with the following working model. Prior to RC formation, nuclear F-actin is induced by one or more immediate early or early gene products, either directly or by modulation of host protein synthesis or function. Following HCMV DNA synthesis at the periphery of RCs, capsid assembly and packaging occur in and/or near RCs within the nuclear interior. Subsequent movement of capsids to the nuclear periphery is a rate-limiting step, as most capsids are present in RC-like inclusions under steady-state conditions. Once capsids are assembled and packaged with viral DNA in RCs, they move to the nuclear periphery via an F-actin-dependent mechanism. While our results are consistent with the proposal that capsid trafficking occurs via a myosin motor protein(s) walking on F-actin, as suggested for alphaherpesviruses (25, 26), our results do not rule out the possibility that actin dynamics propel capsids or other mechanisms (see below). Later in infection, when some nuclear F-actin is oriented along the nuclear rim, capsid movement may occur along the nuclear periphery to “scan” for gaps in the nuclear lamina so that capsids can access the inner nuclear membrane (INM) for primary envelopment. Notably, such a mechanism may be particularly advantageous to HCMV by spatially coordinating nuclear egress with cytoplasmic maturation in the assembly compartment, which forms distinctly at one side of the nucleus (46).

It has been proposed that alphaherpesvirus infection induces enlargements of interchromatin domains so that capsids can move efficiently to the nuclear rim by diffusion, rather than by directed movements dependent on F-actin (28). It is possible that HCMV uses a different mechanism for capsid movement to the nuclear rim than do alphaherpesviruses. Additionally, LatA treatment did not completely ablate capsid accumulation in the cytoplasm or capsid localization away from RC-like inclusions. Although that could be due to incomplete efficacy, it remains possible that some HCMV capsids reach the nuclear periphery by a non-actin-based mechanism such as diffusion. It also remains possible that nuclear F-actin induction plays some role in remodeling nuclear architecture to enable efficient diffusion of HCMV capsids toward the nuclear periphery. Thus, further investigation is required to test whether HCMV capsids undergo directed movement in the nucleus in an F-actin-dependent manner. Regardless, any model to explain herpesvirus capsid movement to the nuclear rim should take the results reported here into account.

While the nuclear cytoskeleton has been implicated in numerous processes in both infected and uninfected cells, its structure and functions are still poorly understood. Thus, how nuclear F-actin is involved in herpesvirus capsid movement remains an important subject in the context of not only virology but also cell biology. Our findings should help pave the way toward a better understanding of both herpesvirus nuclear egress and, more generally, actin-based processes in the nucleus.

## MATERIALS AND METHODS

### Cells and viruses.

Human foreskin fibroblast (HFF) cells (ATCC; CRL-1684) and human embryonic kidney (293T) cells (ATCC; CRL-11268) were propagated in Dulbecco's modified Eagle's medium (DMEM) containing 10% fetal bovine serum (FBS). The HCMV laboratory strain AD169 was used in all experiments unless otherwise stated. AD169-RV encoding FLAG-tagged versions of UL53 (53-F) (47) or UL44 (44-F) (48) was described previously. HSV-1 (KOS) encoding VP26-RFP (49) was a kind gift from Prashant Desai, Johns Hopkins University. Viruses were propagated and titrated as described previously (50).

### Generation of LifeAct-GFP-NLS-expressing HFFs.

To generate HFFs stably expressing LifeAct-GFP-NLS, the LifeAct-GFP-NLS insert was removed from its original vector (gift from Robert Grosse, University of Marburg) and cloned into pLENTI-PGK-Puro vector (Addgene plasmid catalog no. 19068; gift from Eric Campeau). To generate lentiviruses, 5 × 10^5^ 293T cells/well were plated in a 6-well plate and transfected with LifeAct-GFP-NLS lentiviral vector along with psPAX2 (Addgene plasmid catalog no. 12260) and pMDG.2 (catalog no. 12259) packaging plasmids (gifts from Didier Trono) using Roche Xtreme Gene HD transfection reagent (according to the manufacturer’s lentivirus production protocol). Forty-eight hours after transfection, supernatants containing lentiviruses were harvested and centrifuged at 1,000 × *g* for 5 min to remove cellular debris. Lentiviral supernatant was filtered, then mixed with Polybrene (Sigma; final concentration of 8 µg/ml), and then used to transduce 3 × 10^5^ HFFs/well in a 6-well plate. Transduced HFFs were then selected with puromycin (Sigma; final concentration, 1 µg/ml) and expanded.

### Immunofluorescence.

HFFs (1 × 10^5^ per well) stably expressing LifeAct-GFP-NLS were seeded on glass coverslips in a 24-well plate followed by either mock infection or infection with HCMV or HSV-1 (as indicated in the text). At the time points indicated, cells were fixed at room temperature (RT) in 3.7% formaldehyde–Dulbecco’s phosphate-buffered saline (DPBS). Cells were then permeabilized at RT in 0.1% Triton X-100–DPBS, washed 3 times with DPBS, and blocked overnight in a mixture of 1% bovine serum albumin (BSA; Sigma) and 5% human serum (Sigma) in DPBS. The following antibodies and dilutions were used for primary staining: mouse anti-FLAG M2 (Sigma; F1804), 1:500; mouse anti-IE 1/2 (Virusys; P1215), 1:200; mouse anti-major capsid protein (MCP) (gift from William Britt), 1:200. Antibodies were diluted in a mixture of 1% BSA-5% human serum in DPBS and added to coverslips for 1 h at RT with rocking. Primary antibodies were removed, and coverslips were washed 3 times with DPBS for 5 min with rocking at RT. The staining procedure was repeated with the appropriate fluorescently labeled Alexa Fluor secondary antibodies (Invitrogen), and DAPI was applied in the last 10 min of the secondary antibody incubation. After the final washes, coverslips were mounted on glass slides using ProLong antifade reagent (Invitrogen). Imaging was conducted at the Nikon Imaging Center at Harvard Medical School using a Nikon Ti spinning-disk confocal laser microscope equipped with an Orca-AG cooled charge-coupled device (CCD) camera (Hamamatsu). Postacquisition image analysis was conducted using MetaMorph and ImageJ software packages.

### Live-cell imaging.

Nuclear F-actin induction was observed in live cells by infecting HFFs stably expressing LifeAct-GFP-NLS with WT HCMV (MOI of 3). Directly following infection, cells were imaged at the Nikon Imaging Center at Harvard Medical School using a Nikon Ti epifluorescence microscope equipped with an Orca-ER cooled CCD camera (Hamamatsu) and an incubation chamber (OkoLab). Images were taken every 3 min up to 28 hpi.

### Super-resolution microscopy.

LifeAct-GFP-NLS-expressing HFFs were infected with WT HCMV (MOI of 3) and fixed at RT in 3.7% formaldehyde-DPBS at 24 hpi. 3D-SIM data were collected at the Cell Biology Microscopy Facility at Harvard Medical School using a DeltaVision OMX V4 Blaze system (GE Healthcare) equipped with a 60×/1.42-numerical-aperture (NA) Plan Apo oil immersion objective lens (Olympus), 488 solid state laser, and a pco.edge 5.5 scientific complementary metal oxide semiconductor (sCMOS) camera. z-stacks were acquired with a z-step of 125 nm and with 15 raw images per plane (five phases, three angles). Spherical aberration was minimized using immersion oil matching. Super-resolution images were computationally reconstructed from the raw data sets with a channel-specific measured optical transfer function (OTF) and a Wiener filter constant of 0.001 to 0.002 using softWoRx 6.1.3 (GE Healthcare).

### Cellular fractionation and Western blot analysis.

LifeAct-GFP-NLS-expressing HFFs (2 × 10^5^/well) were seeded in a 12-well plate and either mock infected or infected with WT HCMV (MOI of 1). At 72 hpi, cells were separated into nuclear and cytoplasmic fractions using an NE-PER extraction kit (Thermo). For Western blotting, each fraction was mixed with an equal volume of 2× Laemmli buffer (Bio-Rad), boiled at 95°C for 5 min, and run on a 4 to 20% SDS-polyacrylamide gel (Bio-Rad). Proteins were then transferred onto a polyvinylidene difluoride (PVDF) membrane, blocked with 5% milk in DPBS-T (DPBS with 0.5% Tween 20), and probed with primary antibodies overnight at 4°C with rocking. The following antibody dilutions were used: mouse anti-β-actin (Sigma; A5441), 1:5,000; rabbit anti-GFP (Invitrogen; A11122), 1:1,000; goat anti-lamin B (Santa Cruz; 6216), 1:200; rabbit anti-GAPDH (Cell Signaling; 14C10), 1:1,000. Membranes were washed 3 times with DPBS-T for 10 min at room temperature (RT) with rocking. Membranes were then incubated with secondary antibodies conjugated to horseradish peroxidase (HRP) (Southern Biotech) at 1:1,000 for 1 h at RT with rocking, followed by washing. Finally, chemiluminescence solution (Pierce) was added to membranes and signal was detected with film.

### Determinants of nuclear F-actin induction.

HFFs (1 × 10^5^ per well) stably expressing LifeAct-GFP-NLS were seeded on glass coverslips in a 24-well plate. For UV inactivation, 1 ml of thawed WT HCMV virus stock was removed from its storage vial and added to a 25-mm dish. The open dish was placed on ice approximately 12 in. away from a UV lamp for 15 min. Mock UV inactivation was done concurrently using the same procedure except without UV. Cells were then infected with UV-inactivated or mock-UV-inactivated HCMV (MOI of 1), fixed at 24 hpi, and processed for immunofluorescence microscopy as described above.

For drug treatments, cells were infected with WT HCMV (MOI of 1). After 1.5 h, the viral inoculum was replaced with medium containing 50 mg/ml cycloheximide (CHX), 100 µM ganciclovir (GCV), or DMSO (0.5%). At 24 hpi, cells were fixed and processed for immunofluorescence microscopy as described above.

### F-actin inhibitors.

To test the effects of latrunculin A (LatA; Invitrogen) and cytochalasin D (CytoD; Sigma), 1 × 10^5^ HFFs/well stably expressing LifeAct-GFP-NLS were seeded on glass coverslips in a 24-well plate and infected with WT HCMV (MOI of 5) on the following day. At 72 hpi, medium was removed and fresh medium containing LatA or CytoD, or DMSO (0.5%; vehicle control), was added back to cells. The next day (96 hpi), cells were fixed and processed for immunofluorescence assay (IFA) as described above.

To test the effect of LatA on RC formation, LifeAct-GFP-NLS-expressing HFFs were seeded on glass coverslips as described above. Cells were then infected with 44-F HCMV (MOI of 1). After absorption, the inocula were replaced with 8 µM LatA or DMSO (0.5%). At 48 hpi, cells were fixed and processed for immunofluorescence microscopy as described above.

For virus titration and EM assays, 2 × 10^5^ HFFs/well were seeded in a 12-well plate and infected with WT HCMV (MOI of 5) and drugs were added as described above. At 96 hpi, medium was removed and titrated to measure viral production, and the cell monolayers were fixed for EM analysis to measure nuclear egress and intranuclear capsid distribution as described in the text.

### Transmission electron microscopy.

EM was utilized to assess nuclear egress and intranuclear capsid distribution by counting capsids in the cytoplasm and nuclei and within or outside electron-dense RC-like inclusions in representative whole-cell sections under the conditions described above. Processing for image acquisition and data analysis were performed essentially as described previously (51). The Mann-Whitney test was applied to calculate *P* values using GraphPad Prism software (V6.0d).

## SUPPLEMENTAL MATERIAL

Movie S1  Nuclear F-actin induction in live HCMV-infected cells. LifeAct-GFP-NLS-expressing HFFs were infected with WT HCMV (MOI of 3) and imaged with time-lapse fluorescence microscopy from 0 to 28 hpi. Download Movie S1, AVI file, 5 MB

Movie S2  Visualization of nuclear F-actin with super-resolution microscopy. LifeAct-GFP-NLS-expressing HFFs were infected with WT HCMV (MOI of 3), fixed at 24 hpi, and imaged with 3D structured illumination microscopy. Download Movie S2, AVI file, 0.5 MB

Figure S1  Nuclear F-actin colocalizes with capsid protein. LifeAct-GFP-NLS (green)-expressing HFFs were infected with 44-F HCMV (MOI of 1), fixed at 72 hpi, stained with anti-MCP (red) antibodies and DAPI (blue), and imaged with spinning-disk confocal microscopy. Arrows indicate colocalization of nuclear F-actin and MCP. Images are single Z-sections. Bar, 10 µm. Download Figure S1, TIF file, 25.1 MB

Figure S2  Visualization of nuclear F-actin in HSV-1-infected cells. LifeAct-GFP-NLS-expressing HFFs (green) were either mock infected or infected with VP26-RFP (red) HSV-1 (MOI of 3). At 8 hpi, cells were fixed, stained with DAPI (blue), and imaged using spinning-disk confocal microscopy. Images are single Z-sections. Bar, 10 µm. Download Figure S2, TIF file, 25.1 MB

Figure S3  Cellular fractionation. LifeAct-GFP-NLS-expressing HFFs were either mock infected or infected with WT HCMV (MOI of 1). At 72 hpi, cells were separated into nuclear and cytoplasmic fractions and analyzed by Western blotting using the indicated antibodies. Download Figure S3, TIF file, 25.1 MB

Figure S4  Ganciclovir reduces expression of a late viral protein. LifeAct-GFP-NLS-expressing HFFs were infected with HCMV encoding a FLAG-tagged version of the late protein UL53 (MOI of 1) and treated with ganciclovir (GCV) or DMSO (vehicle control) from 0 to 72 hpi. Cells were fixed at 72 hpi, stained with an anti-FLAG antibody (red) and DAPI (blue), and imaged with spinning-disk confocal microscopy. Images are single Z-sections. Bar, 10 µm. Download Figure S4, TIF file, 25.1 MB

Figure S5  Quantification of nuclear F-actin orientations. LifeAct-GFP-NLS (green)-expressing HFFs were infected with 44-F HCMV (MOI of 1), fixed at 72 hpi, stained with an anti-FLAG antibody (red) and DAPI (blue), and imaged with spinning-disk confocal microscopy. The arrows indicate representative nuclear actin filaments in each orientation. The percentage of infected cells containing nuclear F-actin (*n* = 17) was calculated for each type of filament orientation. Images are single Z-sections. Download Figure S5, TIF file, 25.1 MB

Figure S6  Effects of different concentrations of LatA and CytoD on nuclear F-actin. (A) HFFs stably expressing LifeAct-GFP-NLS (green) were infected with WT HCMV (MOI of 5). Medium was removed at 72 hpi and replaced with fresh medium containing LatA, CytoD, or DMSO (control) at the indicated concentrations. Twenty-four hours later (96 hpi), cells were fixed, stained with DAPI (blue), and imaged with spinning-disk confocal microscopy. Images are single Z-sections. Bar, 10 µm. (B) The cells from above were also analyzed by bright-field microscopy to assess morphology. Download Figure S6, TIF file, 25.1 MB

Figure S7  Depolymerization of nuclear F-actin does not affect RC formation or maturation. LifeAct-GFP-NLS (green)-expressing HFFs were infected with 44-F HCMV (MOI of 1) and treated with 8 µM LatA or DMSO vehicle control from 0 to 48 hpi. Cells were then fixed, stained with an anti-FLAG antibody (red) and DAPI (blue), and imaged with spinning-disk confocal microscopy. Images are single Z-sections. Bar, 10 µm. Download Figure S7, TIF file, 25.1 MB

## References

[1] Strang BL 2015 Viral and cellular subnuclear structures in human cytomegalovirus-infected cells. J Gen Virol 96:239–253. doi:10.1099/vir.0.071084-0. 25359764

[2] Mocarski ES, Shenk T, Griffiths P, Pass R 2013 Cytomegaloviruses, p 1960–2014. *In* Knipe DM, Howley PM, Cohen JI, Griffin DE, Lamb RA, Martin MA, Racaniello VR, Roizman B (ed), Fields virology, 6th ed, vol 2 Lippincott Williams & Wilkins, Philadelphia, PA.

[3] Strang BL, Boulant S, Kirchhausen T, Coen DM 2012 Host cell nucleolin is required to maintain the architecture of human cytomegalovirus replication compartments. mBio 3:e01254-16. doi:10.1128/mBio.00301-11. PMC3280463 22318319

[4] Strang BL, Boulant S, Chang L, Knipe DM, Kirchhausen T, Coen DM 2012 Human cytomegalovirus UL44 concentrates at the periphery of replication compartments, the site of viral DNA synthesis. J Virol 86:2089–2095. doi:10.1128/JVI.06720-11. 22156516 PMC3302373

[5] Bender BJ, Coen DM, Strang BL 2014 Dynamic and nucleolin-dependent localization of human cytomegalovirus UL84 to the periphery of viral replication compartments and nucleoli. J Virol 88:11738–11747. doi:10.1128/JVI.01889-14. 25078694 PMC4178712

[6] Penfold ME, Mocarski ES 1997 Formation of cytomegalovirus DNA replication compartments defined by localization of viral proteins and DNA synthesis. Virology 239:46–61. doi:10.1006/viro.1997.8848. 9426445

[7] Borst EM, Wagner K, Binz A, Sodeik B, Messerle M 2008 The essential human cytomegalovirus gene UL52 is required for cleavage-packaging of the viral genome. J Virol 82:2065–2078. doi:10.1128/JVI.01967-07. 18077717 PMC2258901

[8] Giesen K, Radsak K, Bogner E 2000 Targeting of the gene product encoded by ORF UL56 of human cytomegalovirus into viral replication centers. FEBS Lett 471:215–218. doi:10.1016/S0014-5793(00)01407-1. 10767426

[9] Giesen K, Radsak K, Bogner E 2000 The potential terminase subunit of human cytomegalovirus, pUL56, is translocated into the nucleus by its own nuclear localization signal and interacts with importin alpha. J Gen Virol 81:2231–2244. doi:10.1099/0022-1317-81-9-2231. 10950981

[10] Dittmer A, Drach JC, Townsend LB, Fischer A, Bogner E 2005 Interaction of the putative human cytomegalovirus portal protein pUL104 with the large terminase subunit pUL56 and its inhibition by benzimidazole-d-ribonucleosides. J Virol 79:14660–14667. doi:10.1128/JVI.79.23.14660-14667.2005. 16282466 PMC1287559

[11] Borst EM, Bauerfeind R, Binz A, Stephan TM, Neuber S, Wagner K, Steinbrück L, Sodeik B, Lenac Roviš T, Jonjić S, Messerle M 2016 The essential human cytomegalovirus proteins pUL77 and pUL93 are structural components necessary for viral genome encapsidation. J Virol 90:5860–5875. doi:10.1128/JVI.00384-16. 27009952 PMC4907240

[12] Köppen-Rung P, Dittmer A, Bogner E 2016 Intracellular distribution of capsid-associated pUL77 of human cytomegalovirus and interactions with packaging proteins and pUL93. J Virol 90:5876–5885. doi:10.1128/JVI.00351-16. 27053556 PMC4907233

[13] Mettenleiter TC, Müller F, Granzow H, Klupp BG 2013 The way out: what we know and do not know about herpesvirus nuclear egress. Cell Microbiol 15:170–178. doi:10.1111/cmi.12044. 23057731

[14] Funk C, Ott M, Raschbichler V, Nagel C-H, Binz A, Sodeik B, Bauerfeind R, Bailer SM 2015 The herpes simplex virus protein pUL31 escorts nucleocapsids to sites of nuclear egress, a process coordinated by its N-terminal domain. PLoS Pathog 11:e01254-16. doi:10.1371/journal.ppat.1004957. PMC4471197 26083367

[15] Bigalke JM, Heuser T, Nicastro D, Heldwein EE 2014 Membrane deformation and scission by the HSV-1 nuclear egress complex. Nat Commun 5:4131. doi:10.1038/ncomms5131. 24916797 PMC4105210

[16] Klupp BG, Granzow H, Fuchs W, Keil GM, Finke S, Mettenleiter TC 2007 Vesicle formation from the nuclear membrane is induced by coexpression of two conserved herpesvirus proteins. Proc Natl Acad Sci U S A 104:7241–7246. doi:10.1073/pnas.0701757104. 17426144 PMC1855391

[17] Lorenz M, Vollmer B, Unsay JD, Klupp BG, García-Sáez AJ, Mettenleiter TC, Antonin W 2015 A single herpesvirus protein can mediate vesicle formation in the nuclear envelope. J Biol Chem 290:6962–6974. doi:10.1074/jbc.M114.627521. 25605719 PMC4358120

[18] Lee C-P, Liu P-T, Kung H-N, Su M-T, Chua H-H, Chang Y-H, Chang C-W, Tsai C-H, Liu F-T, Chen M-R 2012 The ESCRT machinery is recruited by the viral BFRF1 protein to the nucleus-associated membrane for the maturation of Epstein-Barr virus. PLoS Pathog 8:e01254-16. doi:10.1371/journal.ppat.1002904. PMC3435242 22969426

[19] Bigalke JM, Heldwein EE 2015 Structural basis of membrane budding by the nuclear egress complex of herpesviruses. EMBO J 34:2921–2936. doi:10.15252/embj.201592359. 26511020 PMC4687684

[20] Lye MF, Sharma M, El Omari K, Filman DJ, Schuermann JP, Hogle JM, Coen DM 2015 Unexpected features and mechanism of heterodimer formation of a herpesvirus nuclear egress complex. EMBO J 34:2937–2952. doi:10.15252/embj.201592651. 26511021 PMC4687685

[21] Walzer SA, Egerer-Sieber C, Sticht H, Sevvana M, Hohl K, Milbradt J, Muller YA, Marschall M 2015 Crystal structure of the human cytomegalovirus pUL50-pUL53 core nuclear egress complex provides insight into a unique assembly scaffold for virus-host protein interactions. J Biol Chem 290:27452–27458. doi:10.1074/jbc.C115.686527. 26432641 PMC4645997

[22] Zeev-Ben-Mordehai T, Weberruß M, Lorenz M, Cheleski J, Hellberg T, Whittle C, El Omari K, Vasishtan D, Dent KC, Harlos K, Franzke K, Hagen C, Klupp BG, Antonin W, Mettenleiter TC, Grünewald K 2015 Crystal structure of the herpesvirus nuclear egress complex provides insights into inner nuclear membrane remodeling. Cell Rep 13:2645–2652. doi:10.1016/j.celrep.2015.11.008. 26711332 PMC4700048

[23] Hagen C, Dent KC, Zeev-Ben-Mordehai T, Grange M, Bosse JB, Whittle C, Klupp BG, Siebert CA, Vasishtan D, Bäuerlein FJ, Cheleski J, Werner S, Guttmann P, Rehbein S, Henzler K, Demmerle J, Adler B, Koszinowski U, Schermelleh L, Schneider G, Enquist LW, Plitzko JM, Mettenleiter TC, Grünewald K 2015 Structural basis of vesicle formation at the inner nuclear membrane. Cell 163:1692–1701. doi:10.1016/j.cell.2015.11.029. 26687357 PMC4701712

[24] Chang L, Godinez WJ, Kim I-H, Tektonidis M, de Lanerolle P, Eils R, Rohr K, Knipe DM 2011 Herpesviral replication compartments move and coalesce at nuclear speckles to enhance export of viral late mRNA. Proc Natl Acad Sci U S A 108:E136–E144. doi:10.1073/pnas.1103411108. 21555562 PMC3102408

[25] Feierbach B, Piccinotti S, Bisher M, Denk W, Enquist LW 2006 Alpha-herpesvirus infection induces the formation of nuclear actin filaments. PLoS Pathog 2:e85. doi:10.1371/journal.ppat.0020085. 16933992 PMC1550268

[26] Forest T, Barnard S, Baines JD 2005 Active intranuclear movement of herpesvirus capsids. Nat Cell Biol 7:429–431. doi:10.1038/ncb1243. 15803134

[27] Bosse JB, Virding S, Thiberge SY, Scherer J, Wodrich H, Ruzsics Z, Koszinowski UH, Enquist LW 2014 Nuclear herpesvirus capsid motility is not dependent on F-actin. mBio 5:e01254-16. doi:10.1128/mBio.01909-14. PMC4196236 25293761

[28] Bosse JB, Hogue IB, Feric M, Thiberge SY, Sodeik B, Brangwynne CP, Enquist LW 2015 Remodeling nuclear architecture allows efficient transport of herpesvirus capsids by diffusion. Proc Natl Acad Sci U S A 112:E5725–E5733. doi:10.1073/pnas.1513876112. 26438852 PMC4620878

[29] Riedl J, Crevenna AH, Kessenbrock K, Yu JH, Neukirchen D, Bista M, Bradke F, Jenne D, Holak TA, Werb Z, Sixt M, Wedlich-Soldner R 2008 Lifeact: a versatile marker to visualize F-actin. Nat Methods 5:605–607. doi:10.1038/nmeth.1220. 18536722 PMC2814344

[30] Baarlink C, Wang H, Grosse R 2013 Nuclear actin network assembly by formins regulates the SRF coactivator MAL. Science 340:864–867. doi:10.1126/science.1235038. 23558171

[31] Plessner M, Melak M, Chinchilla P, Baarlink C, Grosse R 2015 Nuclear F-actin formation and reorganization upon cell spreading. J Biol Chem 290:11209–11216. doi:10.1074/jbc.M114.627166. 25759381 PMC4416828

[32] Grosse R, Vartiainen MK 2013 To be or not to be assembled: progressing into nuclear actin filaments. Nat Rev Mol Cell Biol 14:693–697. doi:10.1038/nrm3681. 24088744

[33] Belin BJ, Lee T, Mullins RD, Lappalainen P 2015 DNA damage induces nuclear actin filament assembly by Formin-2 and Spire-1/2 that promotes efficient DNA repair. Elife 4:e01254-16. doi:10.7554/eLife.07735. PMC4577826 26287480

[34] Sharon-Friling R, Shenk T 2014 Human cytomegalovirus pUL37x1-induced calcium flux activates PKCα, inducing altered cell shape and accumulation of cytoplasmic vesicles. Proc Natl Acad Sci U S A 111:E1140–E1148. doi:10.1073/pnas.1402515111. 24616524 PMC3970541

[35] Sharon-Friling R, Goodhouse J, Colberg-Poley AM, Shenk T 2006 Human cytomegalovirus pUL37x1 induces the release of endoplasmic reticulum calcium stores. Proc Natl Acad Sci U S A 103:19117–19122. doi:10.1073/pnas.0609353103. 17135350 PMC1748185

[36] Van Minnebruggen G, Favoreel HW, Jacobs L, Nauwynck HJ 2003 Pseudorabies virus US3 protein kinase mediates actin stress fiber breakdown. J Virol 77:9074–9080. doi:10.1128/JVI.77.16.9074-9080.2003. 12885923 PMC167205

[37] Simpson-Holley M, Colgrove RC, Nalepa G, Harper JW, Knipe DM 2005 Identification and functional evaluation of cellular and viral factors involved in the alteration of nuclear architecture during herpes simplex virus 1 infection. J Virol 79:12840–12851. doi:10.1128/JVI.79.20.12840-12851.2005. 16188986 PMC1235858

[38] Pogoda M, Bosse JB, Wagner FM, Schauflinger M, Walther P, Koszinowski UH, Ruzsics Z 2012 Characterization of conserved region 2-deficient mutants of the cytomegalovirus egress protein pM53. J Virol 86:12512–12524. doi:10.1128/JVI.00471-12. 22993161 PMC3497646

[39] Tandon R, Mocarski ES, Conway JF 2015 The A, B, Cs of herpesvirus capsids. Viruses 7:899–914. doi:10.3390/v7030899. 25730559 PMC4379554

[40] Reference deleted.

[41] Goley ED, Ohkawa T, Mancuso J, Woodruff JB, D’Alessio JA, Cande WZ, Volkman LE, Welch MD 2006 Dynamic nuclear actin assembly by Arp2/3 complex and a baculovirus WASP-like protein. Science 314:464–467. doi:10.1126/science.1133348. 17053146

[42] Coué M, Brenner SL, Spector I, Korn ED 1987 Inhibition of actin polymerization by latrunculin A. FEBS Lett 213:316–318. doi:10.1016/0014-5793(87)81513-2. 3556584

[43] Flanagan MD, Lin S 1980 Cytochalasins block actin filament elongation by binding to high affinity sites associated with F-actin. J Biol Chem 255:835–838. 7356663

[44] Schoenenberger C-A, Buchmeier S, Boerries M, Sütterlin R, Aebi U, Jockusch BM 2005 Conformation-specific antibodies reveal distinct actin structures in the nucleus and the cytoplasm. J Struct Biol 152:157–168. doi:10.1016/j.jsb.2005.09.003. 16297639

[45] Fuchsova B, Serebryannyy LA, de Lanerolle P 2015 Nuclear actin and myosins in adenovirus infection. Exp Cell Res 338:170–182. doi:10.1016/j.yexcr.2015.07.025. 26226218 PMC4624525

[46] Alwine JC 2012 The human cytomegalovirus assembly compartment: a masterpiece of viral manipulation of cellular processes that facilitates assembly and egress. PLoS Pathog 8:e01254-16. doi:10.1371/journal.ppat.1002878. PMC3447744 23028305

[47] Sharma M, Kamil JP, Coughlin M, Reim NI, Coen DM 2014 Human cytomegalovirus UL50 and UL53 recruit viral protein kinase UL97, not protein kinase C, for disruption of nuclear lamina and nuclear egress in infected cells. J Virol 88:249–262. doi:10.1128/JVI.02358-13. 24155370 PMC3911691

[48] Strang BL, Boulant S, Coen DM 2010 Nucleolin associates with the human cytomegalovirus DNA polymerase accessory subunit UL44 and is necessary for efficient viral replication. J Virol 84:1771–1784. doi:10.1128/JVI.01510-09. 20007282 PMC2812382

[49] Desai P, Person S 1998 Incorporation of the green fluorescent protein into the herpes simplex virus type 1 capsid. J Virol 72:7563–7568. 9696854 10.1128/jvi.72.9.7563-7568.1998 PMC110002

[50] Leigh KE, Sharma M, Mansueto MS, Boeszoermenyi A, Filman DJ, Hogle JM, Wagner G, Coen DM, Arthanari H 2015 Structure of a herpesvirus nuclear egress complex subunit reveals an interaction groove that is essential for viral replication. Proc Natl Acad Sci U S A 112:9010–9015. doi:10.1073/pnas.1511140112. 26150520 PMC4517201

[51] Reim NI, Kamil JP, Wang D, Lin A, Sharma M, Ericsson M, Pesola JM, Golan DE, Coen DM 2013 Inactivation of retinoblastoma protein does not overcome the requirement for human cytomegalovirus UL97 in lamina disruption and nuclear egress. J Virol 87:5019–5027. doi:10.1128/JVI.00007-13. 23427156 PMC3624322

