# Supplementary figures and images for: A Role for Nuclear F-Actin Induction in Human Cytomegalovirus Nuclear Egress
Source: mBio. 2016 Aug 23;7(4):e01254-16. doi: 10.1128/mBio.01254-16 (PMC4999551; doi:10.1128/mBio.01254-16)

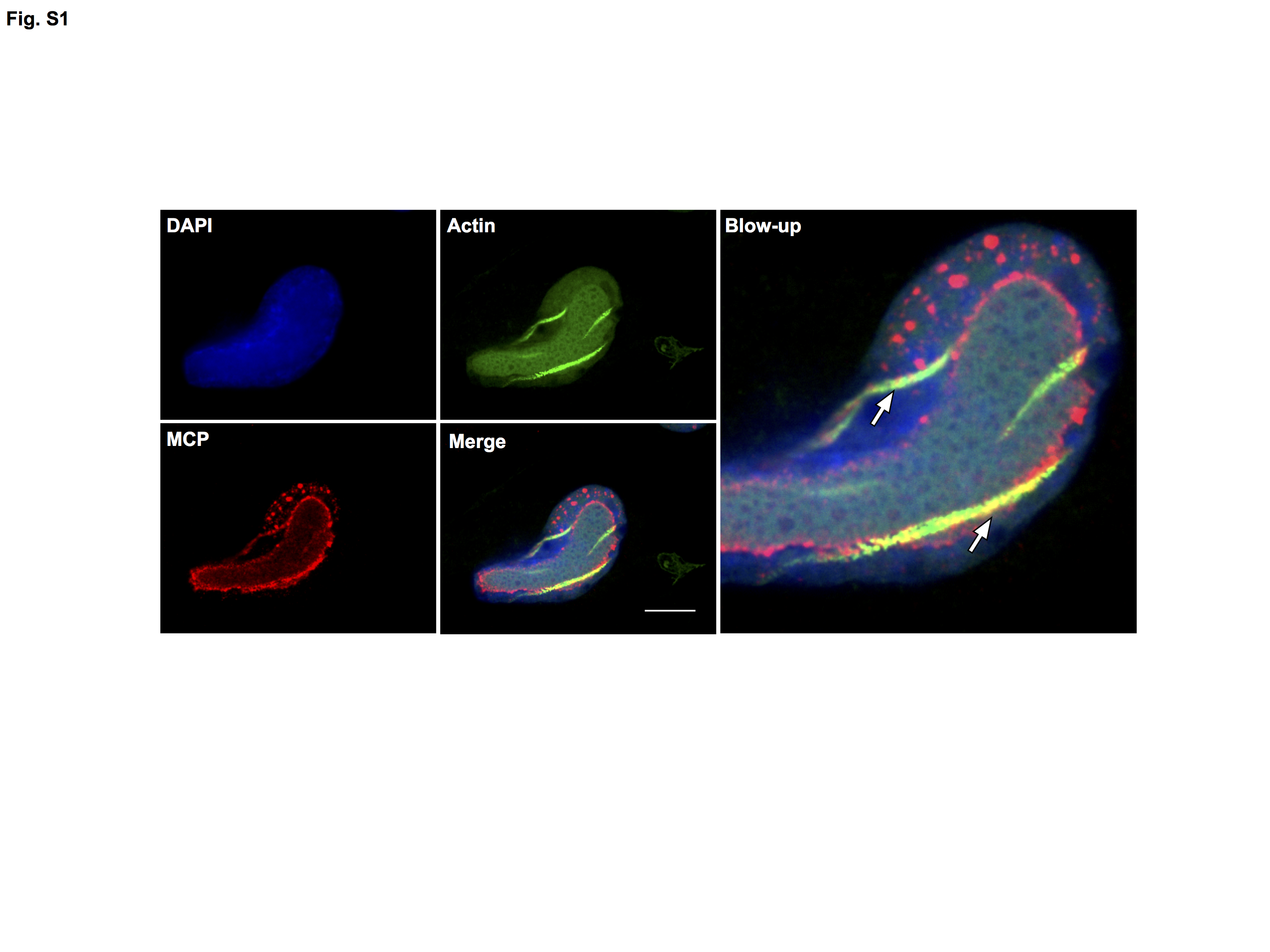

Supplement: Figure S1 — Nuclear F-actin colocalizes with capsid protein. LifeAct-GFP-NLS (green)-expressing HFFs were infected with 44-F HCMV (MOI of 1), fixed at 72 hpi, stained with anti-MCP (red) antibodies and DAPI (blue), and imaged with spinning-disk confocal microscopy. Arrows indicate colocalization of nuclear F-actin and MCP. Images are single Z-sections. Bar, 10 µm. Download [file mbo004162950sf1.tif]

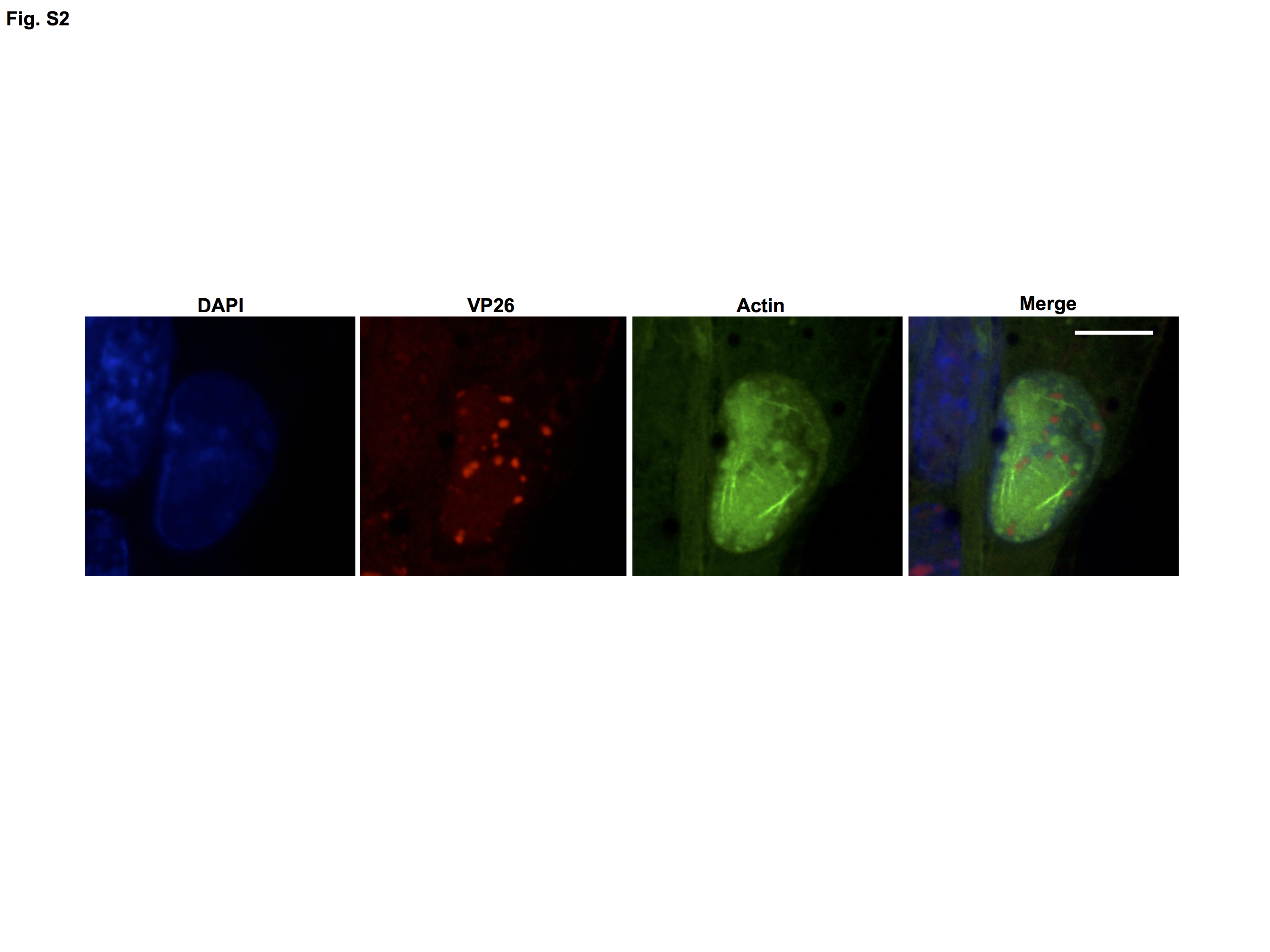

Supplement: Figure S2 — Visualization of nuclear F-actin in HSV-1-infected cells. LifeAct-GFP-NLS-expressing HFFs (green) were either mock infected or infected with VP26-RFP (red) HSV-1 (MOI of 3). At 8 hpi, cells were fixed, stained with DAPI (blue), and imaged using spinning-disk confocal microscopy. Images are single Z-sections. Bar, 10 µm. Download [file mbo004162950sf2.tif]

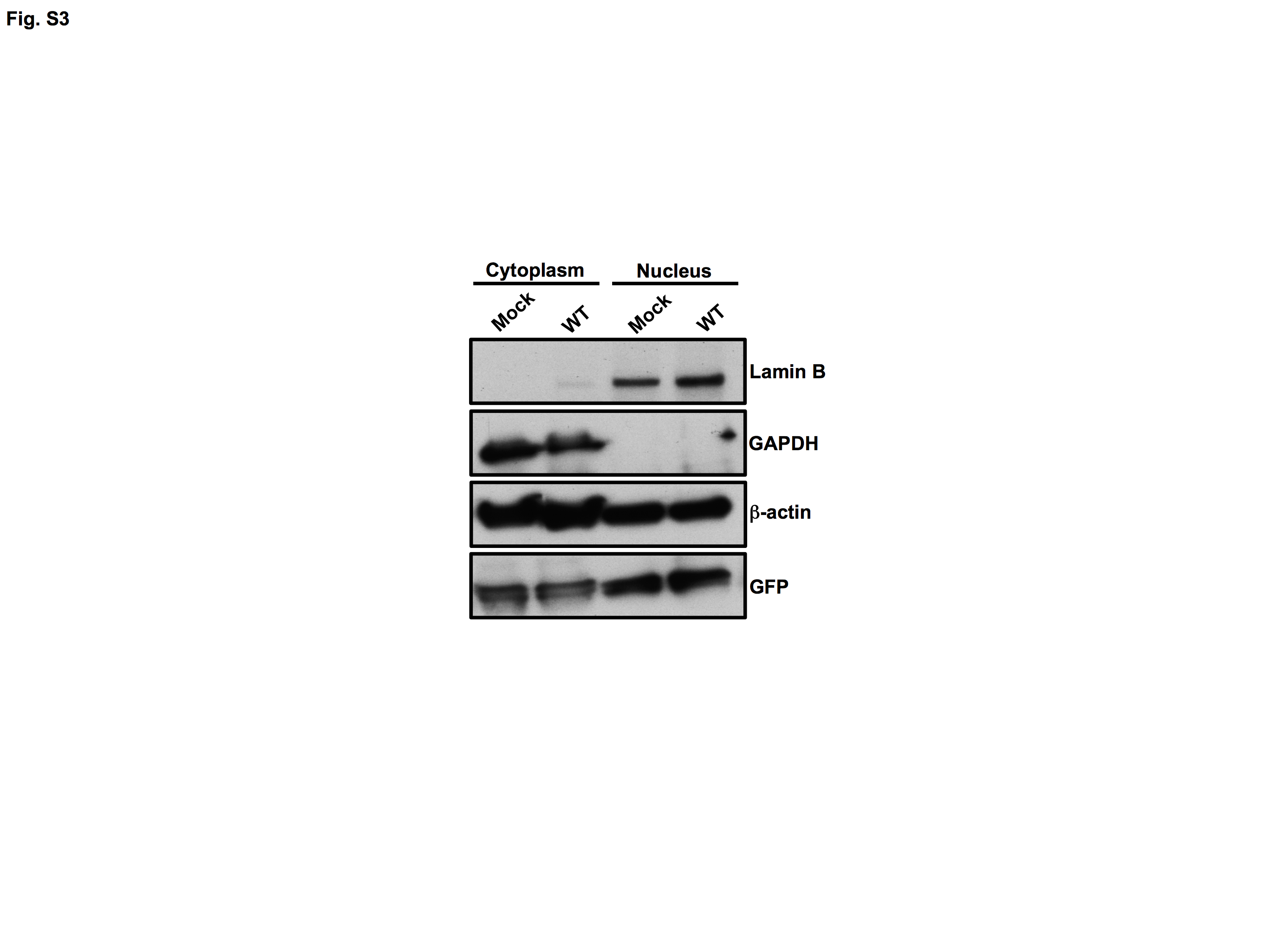

Supplement: Figure S3 — Cellular fractionation. LifeAct-GFP-NLS-expressing HFFs were either mock infected or infected with WT HCMV (MOI of 1). At 72 hpi, cells were separated into nuclear and cytoplasmic fractions and analyzed by Western blotting using the indicated antibodies. Download [file mbo004162950sf3.tif]

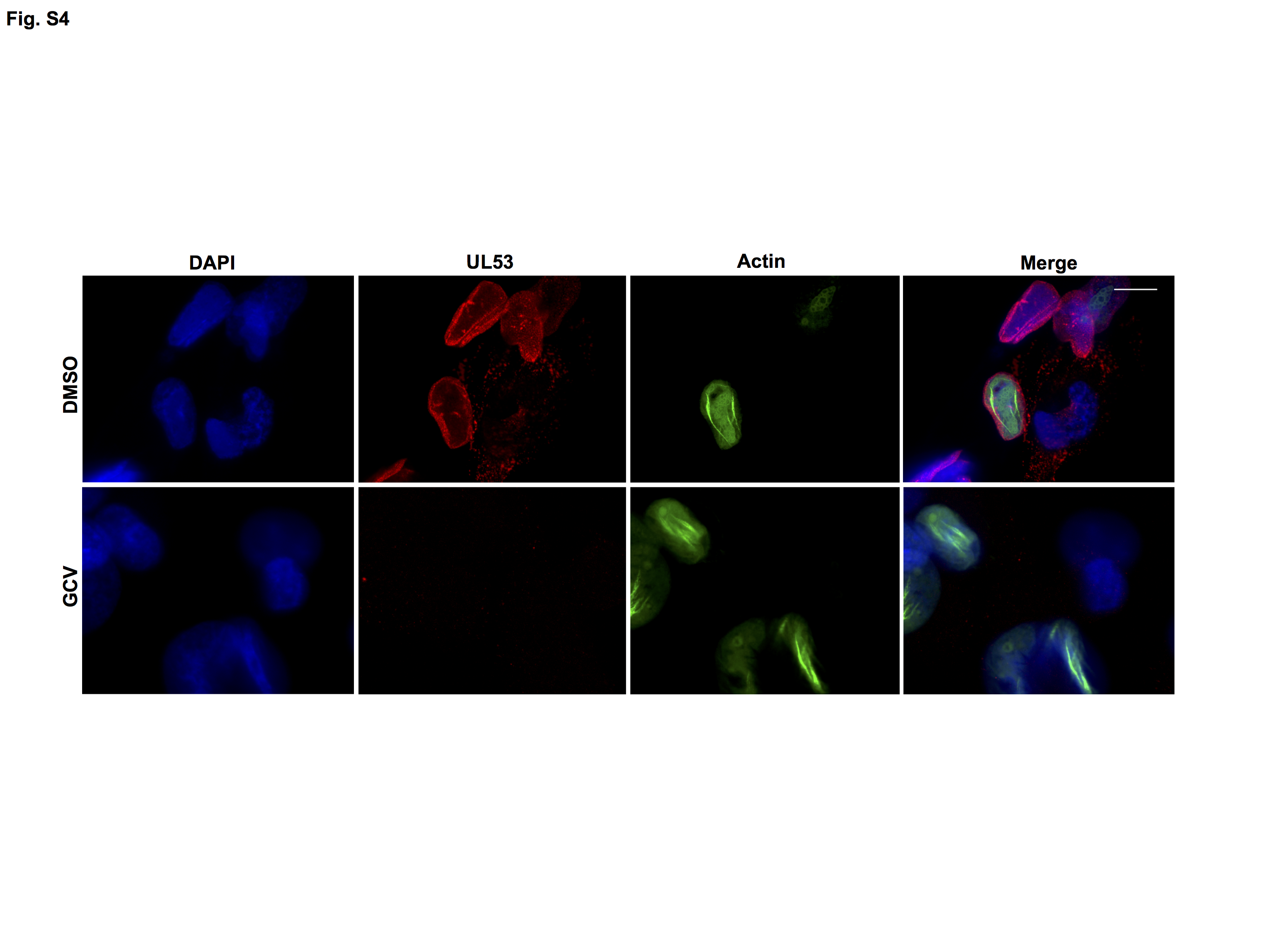

Supplement: Figure S4 — Ganciclovir reduces expression of a late viral protein. LifeAct-GFP-NLS-expressing HFFs were infected with HCMV encoding a FLAG-tagged version of the late protein UL53 (MOI of 1) and treated with ganciclovir (GCV) or DMSO (vehicle control) from 0 to 72 hpi. Cells were fixed at 72 hpi, stained with an anti-FLAG antibody (red) and DAPI (blue), and imaged with spinning-disk confocal microscopy. Images are single Z-sections. Bar, 10 µm. Download [file mbo004162950sf4.tif]

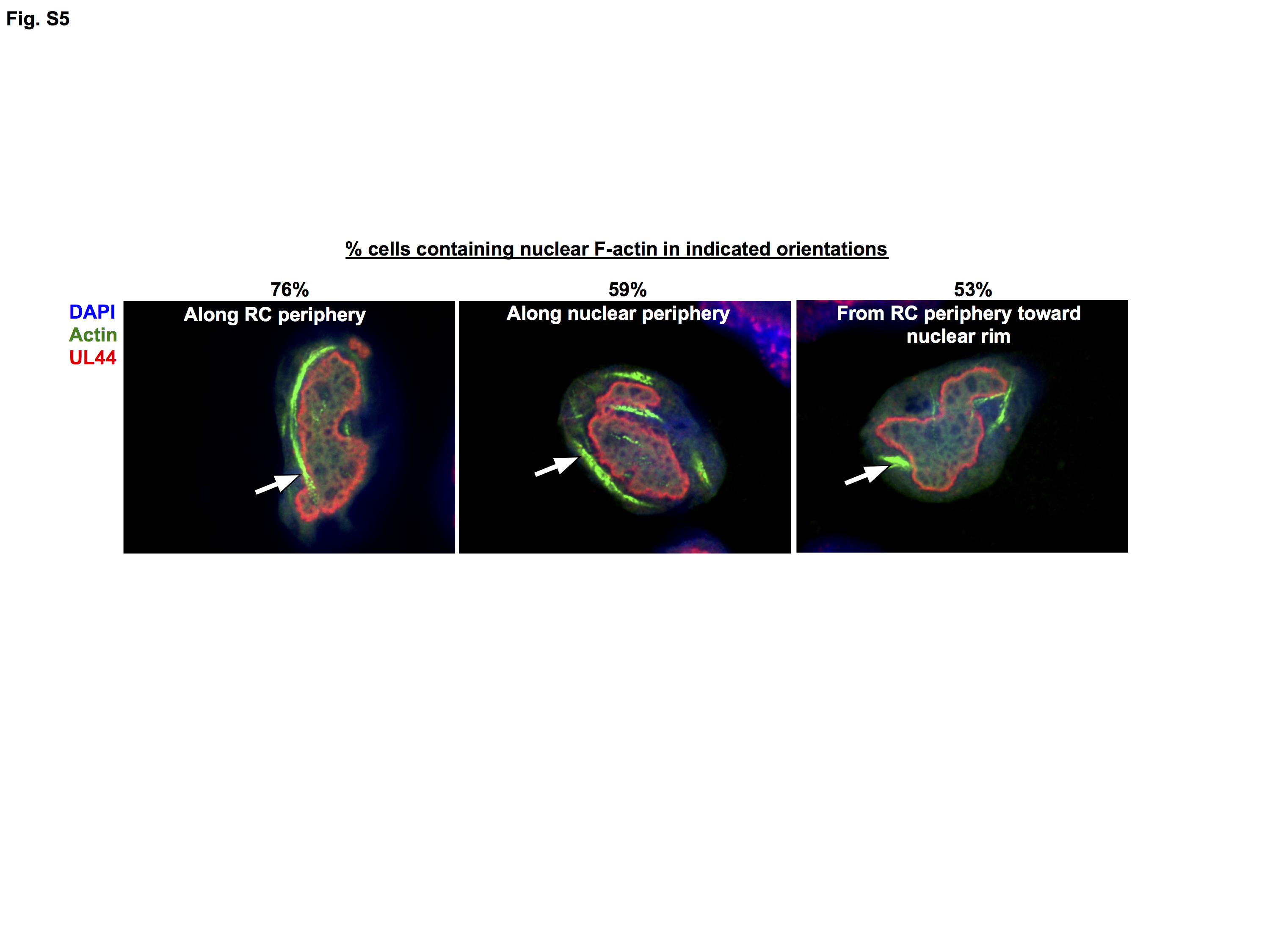

Supplement: Figure S5 — Quantification of nuclear F-actin orientations. LifeAct-GFP-NLS (green)-expressing HFFs were infected with 44-F HCMV (MOI of 1), fixed at 72 hpi, stained with an anti-FLAG antibody (red) and DAPI (blue), and imaged with spinning-disk confocal microscopy. The arrows indicate representative nuclear actin filaments in each orientation. The percentage of infected cells containing nuclear F-actin (n = 17) was calculated for each type of filament orientation. Images are single Z-sections. Download [file mbo004162950sf5.tif]

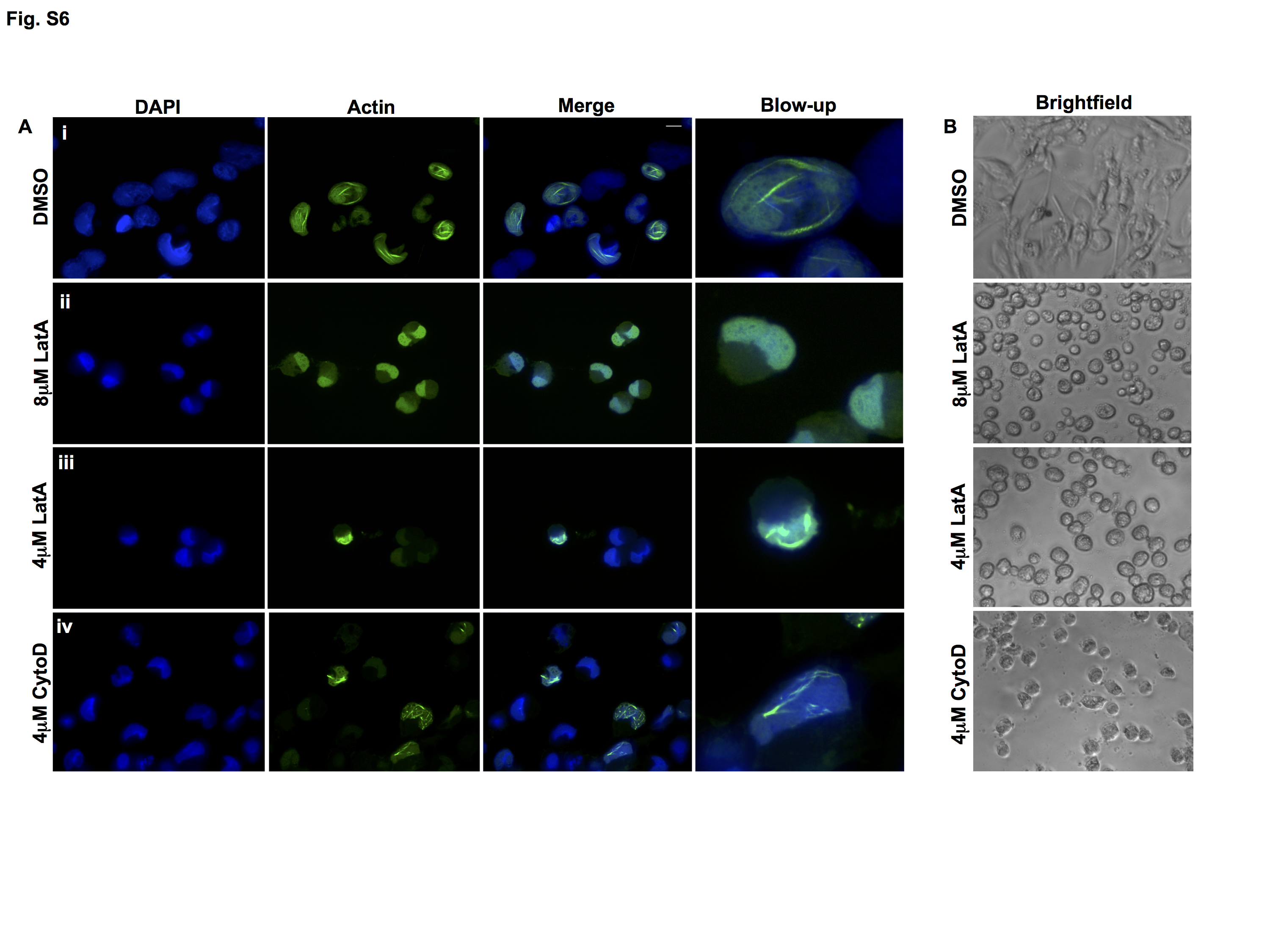

Supplement: Figure S6 — Effects of different concentrations of LatA and CytoD on nuclear F-actin. (A) HFFs stably expressing LifeAct-GFP-NLS (green) were infected with WT HCMV (MOI of 5). Medium was removed at 72 hpi and replaced with fresh medium containing LatA, CytoD, or DMSO (control) at the indicated concentrations. Twenty-four hours later (96 hpi), cells were fixed, stained with DAPI (blue), and imaged with spinning-disk confocal microscopy. Images are single Z-sections. Bar, 10 µm. (B) The cells from above were also analyzed by bright-field microscopy to assess morphology. Download [file mbo004162950sf6.tif]

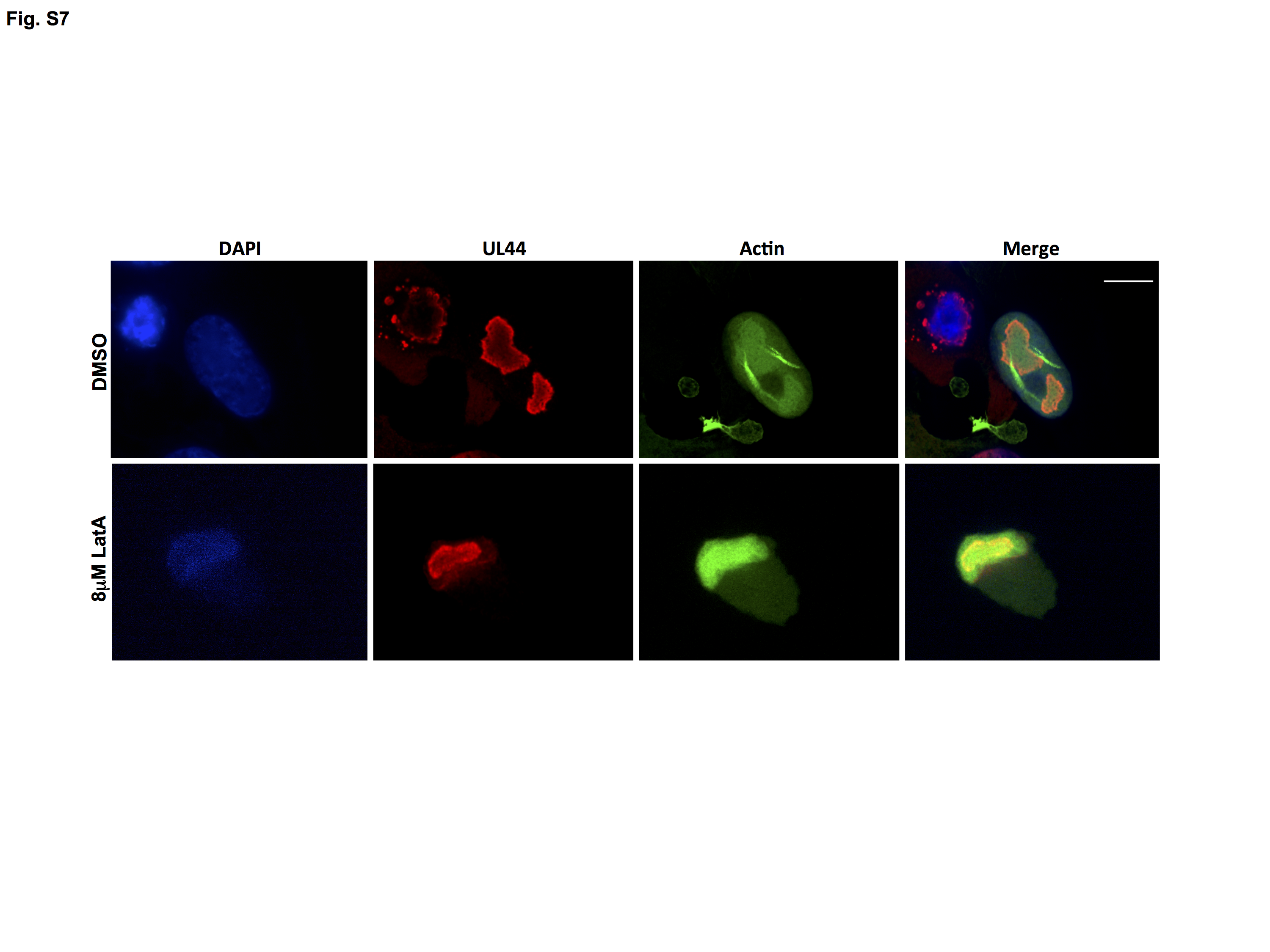

Supplement: Figure S7 — Depolymerization of nuclear F-actin does not affect RC formation or maturation. LifeAct-GFP-NLS (green)-expressing HFFs were infected with 44-F HCMV (MOI of 1) and treated with 8 µM LatA or DMSO vehicle control from 0 to 48 hpi. Cells were then fixed, stained with an anti-FLAG antibody (red) and DAPI (blue), and imaged with spinning-disk confocal microscopy. Images are single Z-sections. Bar, 10 µm. Download [file mbo004162950sf7.tif]
